# Author Correction: Histone 4 lysine 5/12 acetylation enables developmental plasticity of *Pristionchus* mouth form

**DOI:** 10.1038/s41467-023-38693-1

**Published:** 2023-05-18

**Authors:** Michael S. Werner, Tobias Loschko, Thomas King, Shelley Reich, Tobias Theska, Mirita Franz-Wachtel, Boris Macek, Ralf J. Sommer

**Affiliations:** 1 Department for Integrative Evolutionary Biology, Max Planck Institute for Biology Tübingen, Tübingen, 72076 Germany; 2 School of Biological Sciences, The University of Utah, Salt Lake City, UT USA; 3 Proteome Center Tübingen, University of Tübingen, Tübingen, 72076 Germany

**Keywords:** Epigenetic memory, Histone post-translational modifications, Evolutionary developmental biology

Correction to: *Nature Communications* 10.1038/s41467-023-37734-z, published online 13 April 2023

The original version of this Article contained an error in Figure 3a. The color key indicated bars representing DMSO treatment were coloured light purple rather than gray. The legend to Figure 3 inadvertently contains references to colors for panels h-j that are not present in the final figure.

In the caption to Figure 2, the sentence “-values for H4K5ac…” should have read “*P*-values for H4K5ac…”

In the sentence on page 5 of this Article beginning “H4K12ac peaks were significantly enriched…” the term “TSA-induced genes” should have read “genes adjacent to hyperacetylated regions.” In the sentence on page 7 beginning “BRD4 promotes estrogen receptor-positive…” the text “human cell lines^59,60^. ^36^” should have read “human cell lines^36,59,60^.”

In the sentence on page 9 of this Article beginning “Gravid adult P. pacificus were…” the reference “Werner et al.” should have read “Werner et al. 2017^23^.”

In the sentences on pages 9 and 11 beginning “For nematodes, crude nuclei…” “Nuclear fractionation, chromatin…” and “Chromatin state comparisons were…” the references to “Werner et al.” should have read “Werner et al. 2018^40^.”

This has been corrected in the PDF and HTML versions of the Article.

