# Supplementary material for: Histone 4 lysine 5/12 acetylation enables developmental plasticity of Pristionchus mouth form
Source: Nat Commun. 2023 Apr 13;14:2095. doi: 10.1038/s41467-023-37734-z (PMC10102330; doi:10.1038/s41467-023-37734-z)

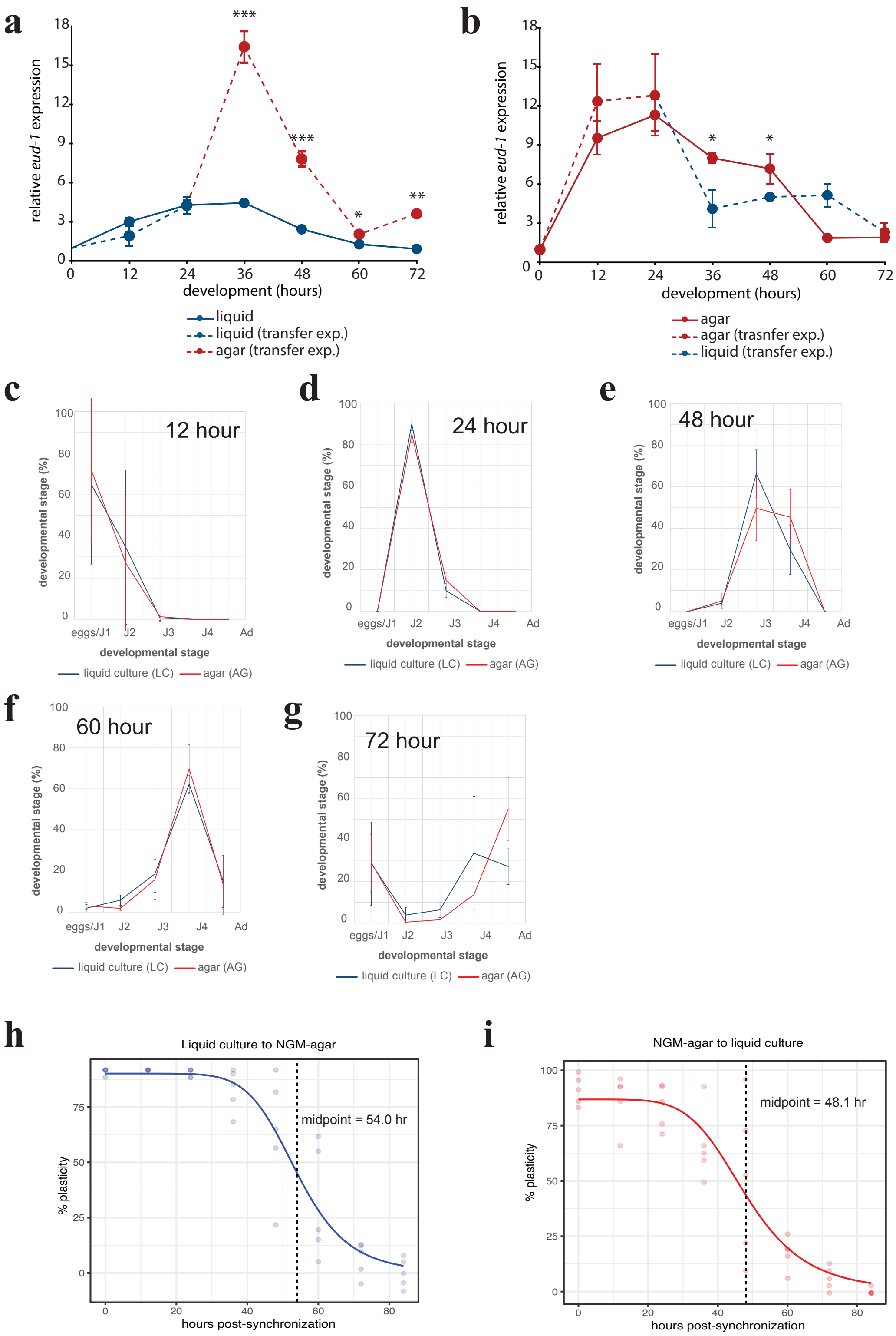

Supplementary Figure 1: Switch gene transcription corresponds to environmental sensitivity. a, Transcription of *eud-1* when worms are transferred from liquid culture to NGM-agar, or **b**, NGM-agar to liquid culture. Relative *eud-1* expression was measured by RT-qPCR (geometric mean of $2^{\Delta Ct}$ relative to *Ppa-cdc-42* and *Ppa-y45F10D.470*, normalized to t'=0). Error bars represent S.E.M. for 3 ind. worm populations, except 'liquid culture 12 hrs' and 'agar-liquid transfer 36 hrs' where n=4, and 'agar-liquid transfer 48 hrs' where n=5. Statistical significance was determined by a 1-sided student's t-test. p values for panel 'a': 36-hr=0.00041, 48-hr=0.00063, 60-hr=0.045, 72-hr=0.0011; p values for panel 'b': 36-hr=0.042, 48-hr=0.047. '*'=p<0.05, '**'=p<0.01, '***'=p<0.001. **c-g**, Developmental stages in agar (red) and liquid culture (blue) after hypochlorite synchronization, n=3 biological replicates, error bars reflect standard deviation centered around the mean. **h**, logistic fit of reciprocal transplant experiments (Fig. 1c) from liquid to NGM-agar and **i**, vice-versa. Source data are provided as a Source Data file.

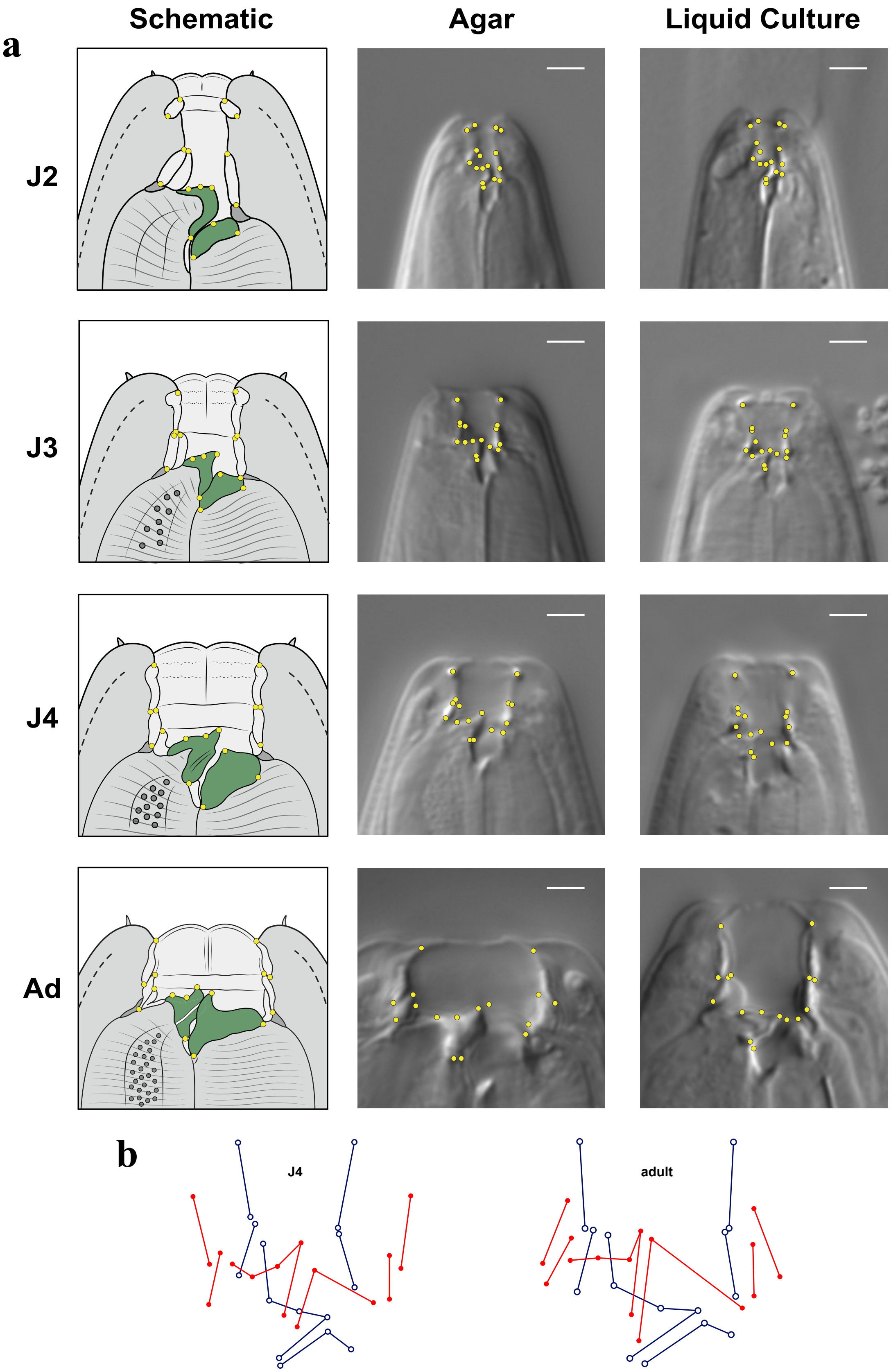

Supplementary Figure 2: Homologous landmarks for quantitative geometric morphometrics. a, Homologous structural landmarks used for GM in both NGM-agar and liquid culture at each developmental stage (except for J1). Note, only one morph (Eu) is shown for the adult illustration, and some landmarks in the microscopy images are out of the current focal plane. Scale bar = 5 µm. Schematics drawn in Adobe illustrator, and images taken using 100x 1.4 oil immersion objective with DIC prism on a Zeiss Axio Imager. **b**, Wire-frame plots of each morph (red=Eu, blue=St) in J4 and adults. Produced in R according to Theska et al. 2020. Source data are provided as a Source Data file.

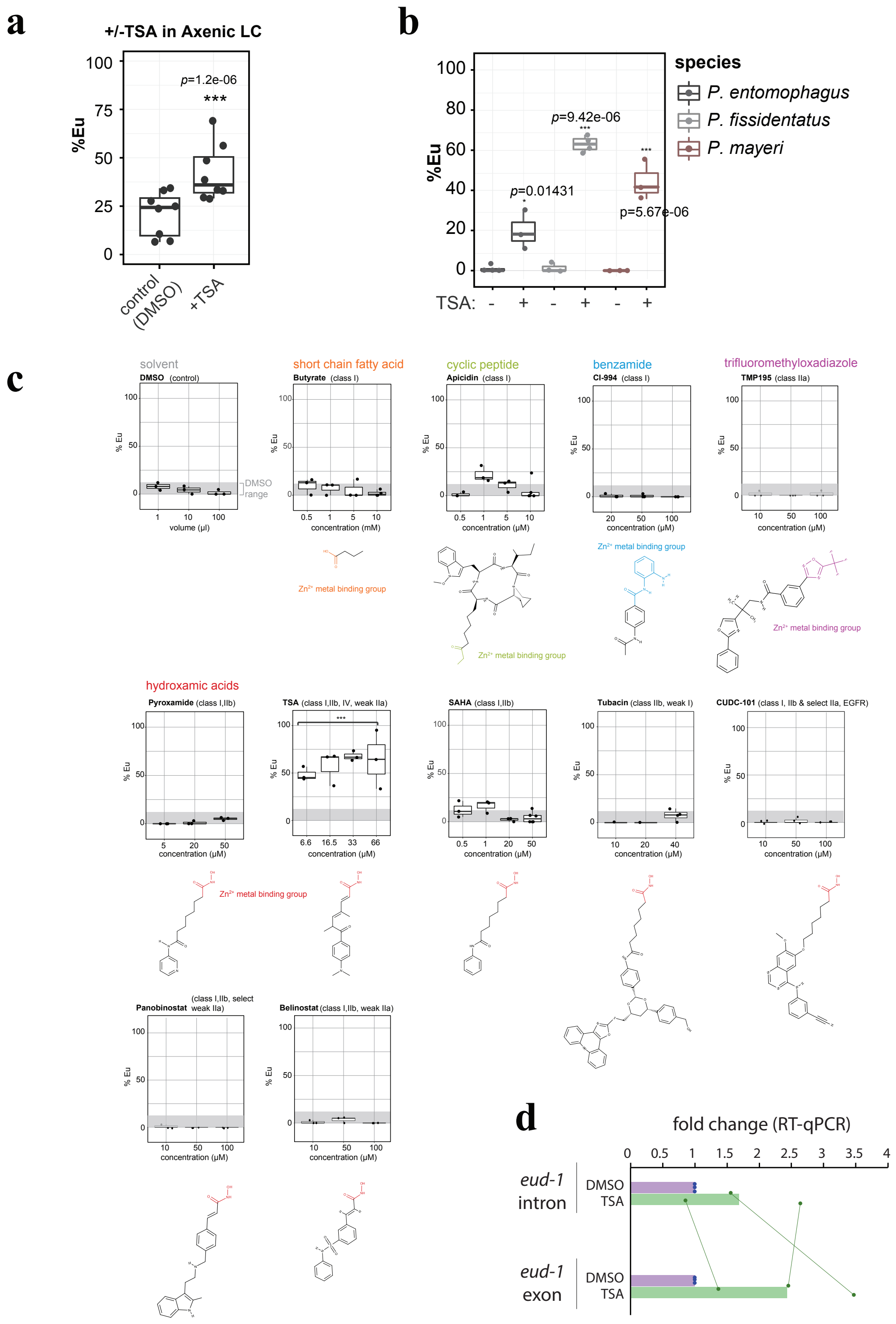

Supplementary Figure 3: TSA has a conserved and specific effect on mouth form. a, Phenotype of *P. pacificus* in axenic liquid culture +/- TSA, n=8 biologically independent worm populations. **b**, Different Pristionchus species in LC +/- TSA, n=7 biologically independent *P. fissidenatus* populations (3 DMSO, 4 TSA), 7 *P. entomophagus* populations (4 DMSO, 3 TSA), and 6 *P. mayeri* populations (3 each). **c**, Phenotype of *P. pacificus* animals grown in liquid culture with the indicated amounts of HDAC inhibitors. Samples without TSA were treated with 100 µl DMSO, n≥3 for all concentrations. Statistical significance in 'a-c' was calculated by glm relative to DMSO. Box-plot minima and maxima represent the 25% and 75% quantile, respectively, and middle bars represent the 50% quantile (median). Whiskers denote 1.5x the inter-quartile range. p value for TSA = 1.42e-09. '*'=p<0.05, '**'=p<0.01, '***'=p<0.001. **d**, RT-qPCR of *eud-1* intronic and exonic segments +/- TSA. qPCR was performed with distinct intronic and exonic primers and normalized to the geometric mean of *Ppa-cdc-42* and *Ppa-Y45F10D.470*. Bar plot indicates avg. fold-change between replicates, n=3. Lines connecting data points indicate paired replicates. Source data are provided as a Source Data file.

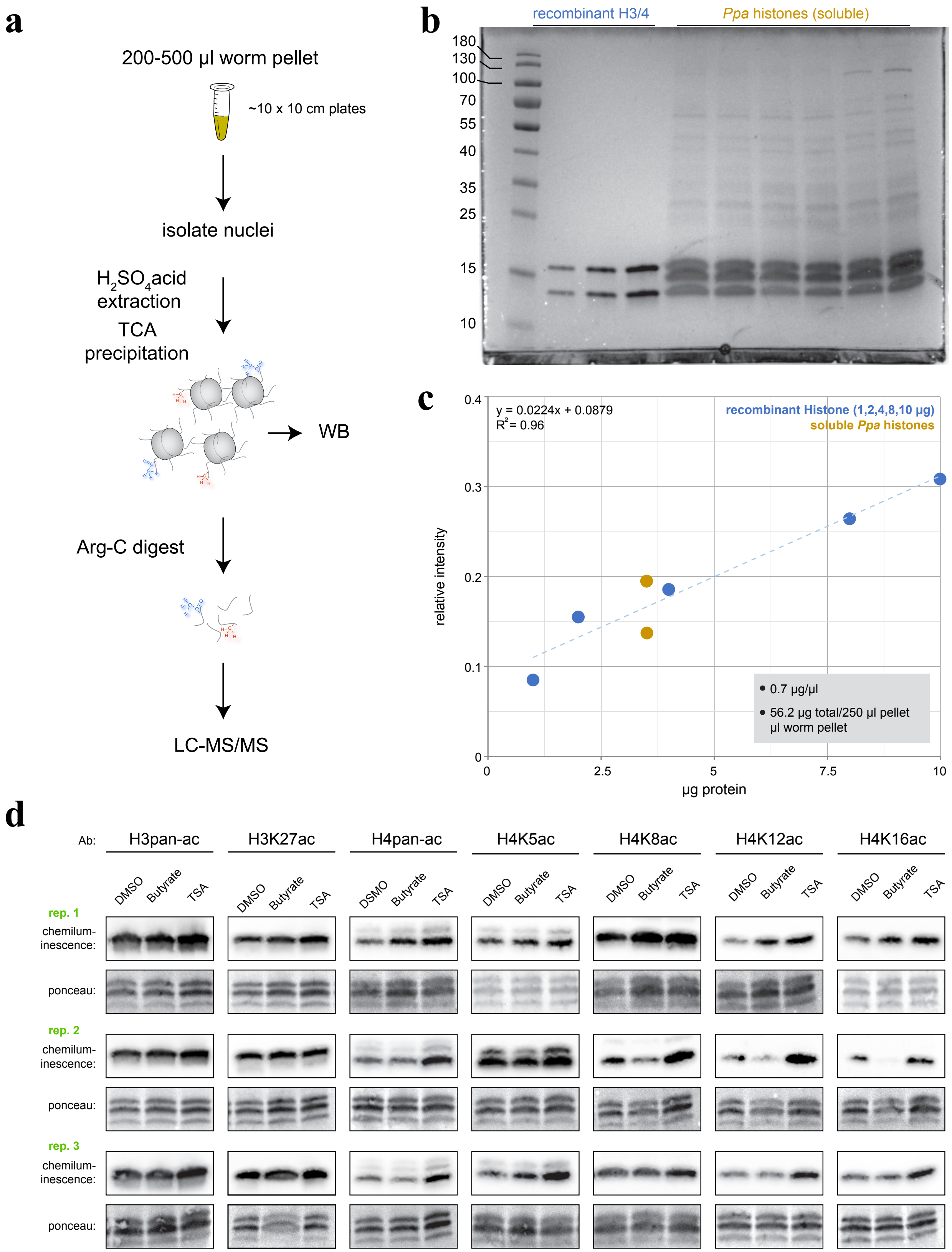

Supplementary Figure 4: **Histone Purification and Raw Data for Western Blots. a**, Schematic of histone acid extraction for Western Blot and LC-MS/MS after digestion with Arg-C protease. **b**, Example SDS-PAGE of histone extraction and a recombinant H3/4 calibration curve. Proteins were visualized with Coomassie Brilliant Blue R-250. **c**, Example calibration curve with recombinant histone used to calculate *P. pacificus* histone quantity for WB and LC-MS/MS, n=2 technical replicates. **d**, Raw data of chemiluminescent signal from three independent biological replicate WBs for each antibody, and total histone amount transferred to nitrocellulose membranes visualized with Ponceau Red. Source data are provided as a Source Data file.

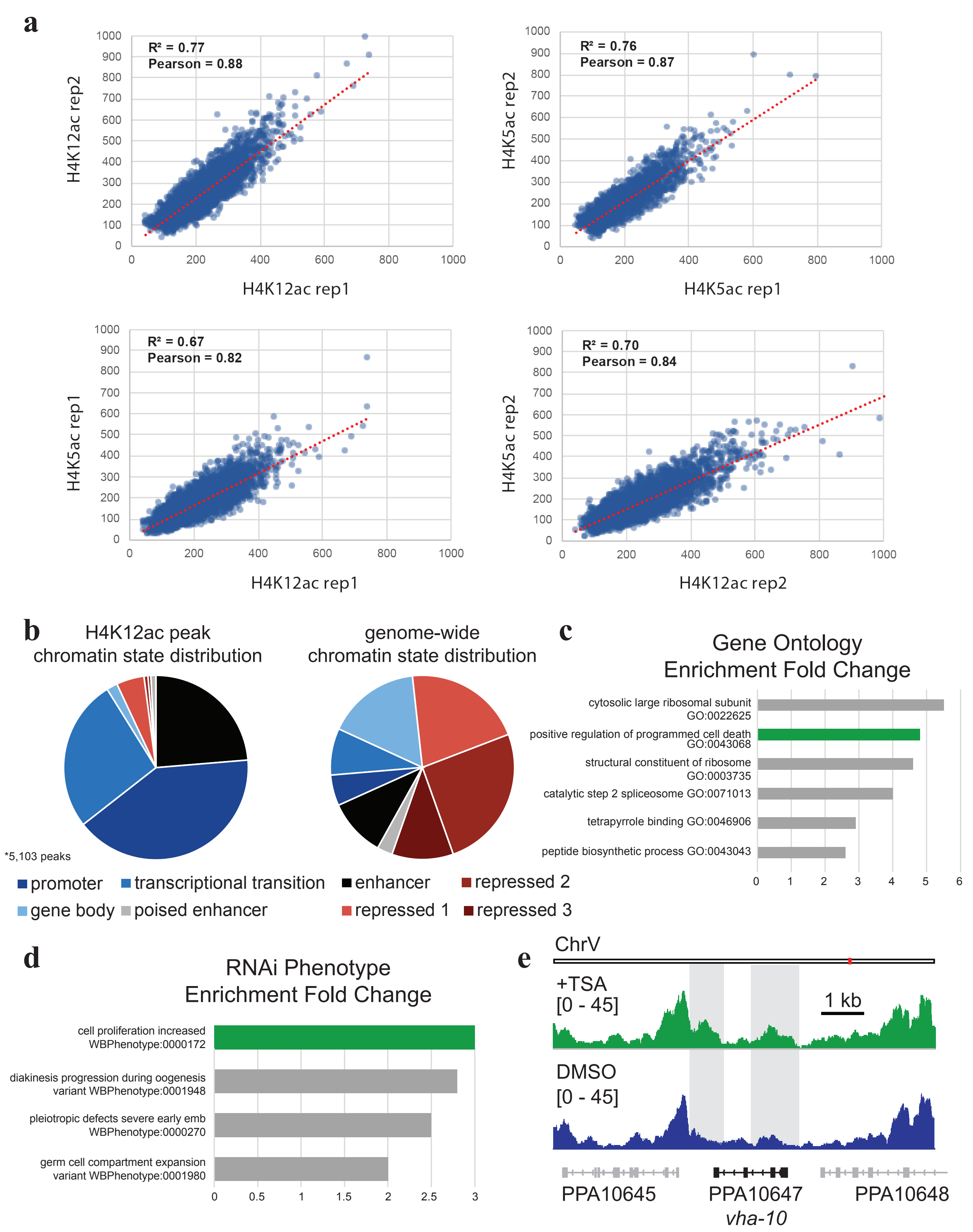

Supplementary Figure 5: Analysis of H4K5/12ac +/- TSA. a, ChIP-seq tag correlations between H4K5 and 12 acetylation at H4K12ac peaks. **b**, Distribution of H4K12ac peaks in each chromatin state, compared to genome-wide distributions, from Werner et al., 2018. Peaks were significantly enriched in regulatory states (promoter, enhancer and transcriptional transition; Fisher's Exact Test). **c-d**, Gene Set Enrichment Analysis of *C. elegans* best-hit homologs (WormBase). Bar plots represent significant observed vs. expected genes in Gene Ontology (c) and RNAi phenotypes (d). **e**, Example gene (*Ppa-vha-10*) with 'programed cell death' ontology. Source data are provided as a Source Data file.

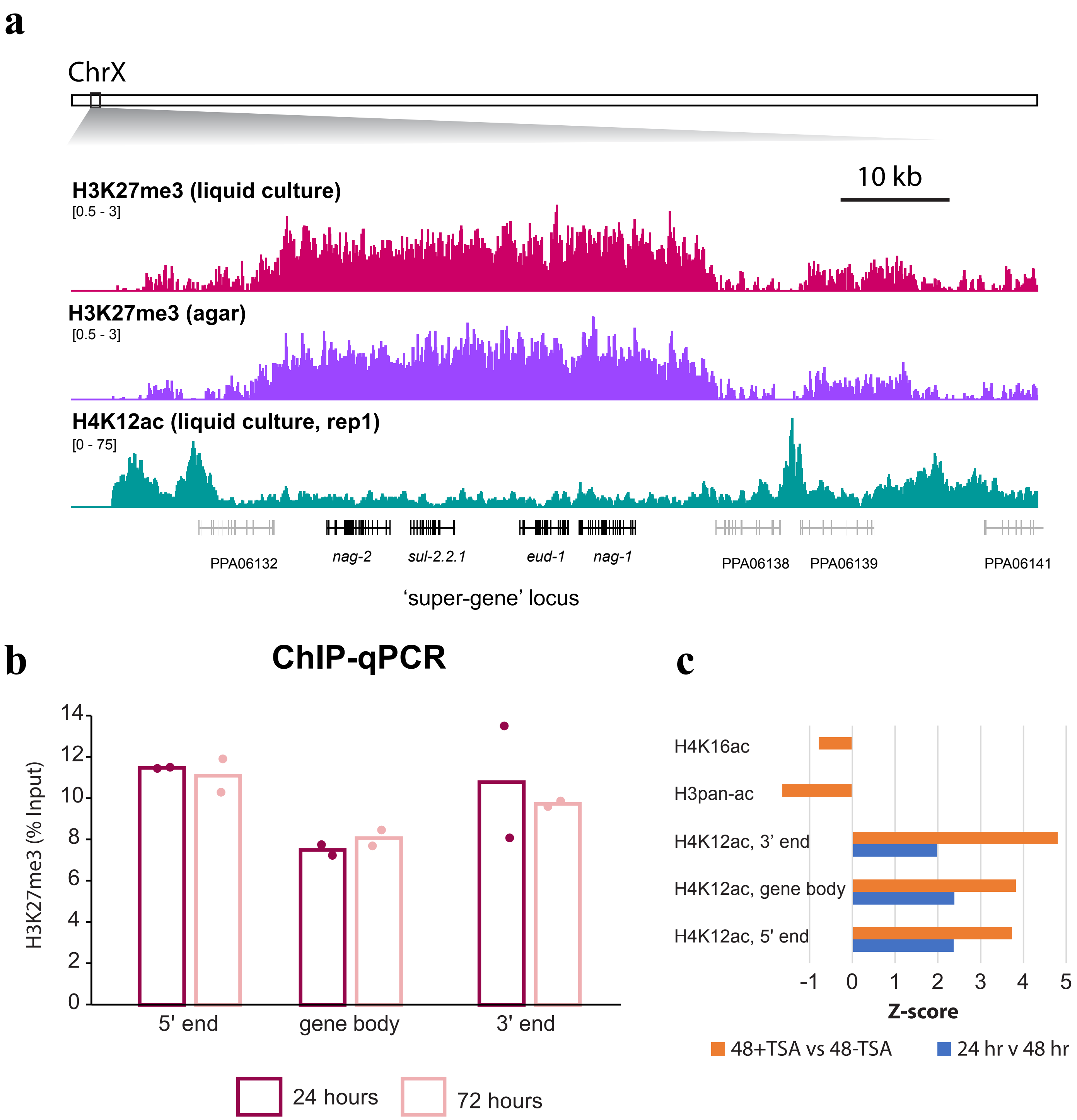

Supplementary Figure 6: H3K27me3 chromatin immunoprecipitation. a, ChIP-seq of H3K27me3 from 48-hour worms grown in liquid culture, compared to H4K12ac in the same conditions. **b**, ChIP-qPCR of H3K27me3 at 24 hours and 72 hours across *eud-1* (same loci probed in main text figure 3), n = 2 biological replicates. Differences were not significant, Student's t-test. **c**, Z-scores of ChIP-qPCR data from Fig. 3h-k. Source data are provided as a Source Data file.

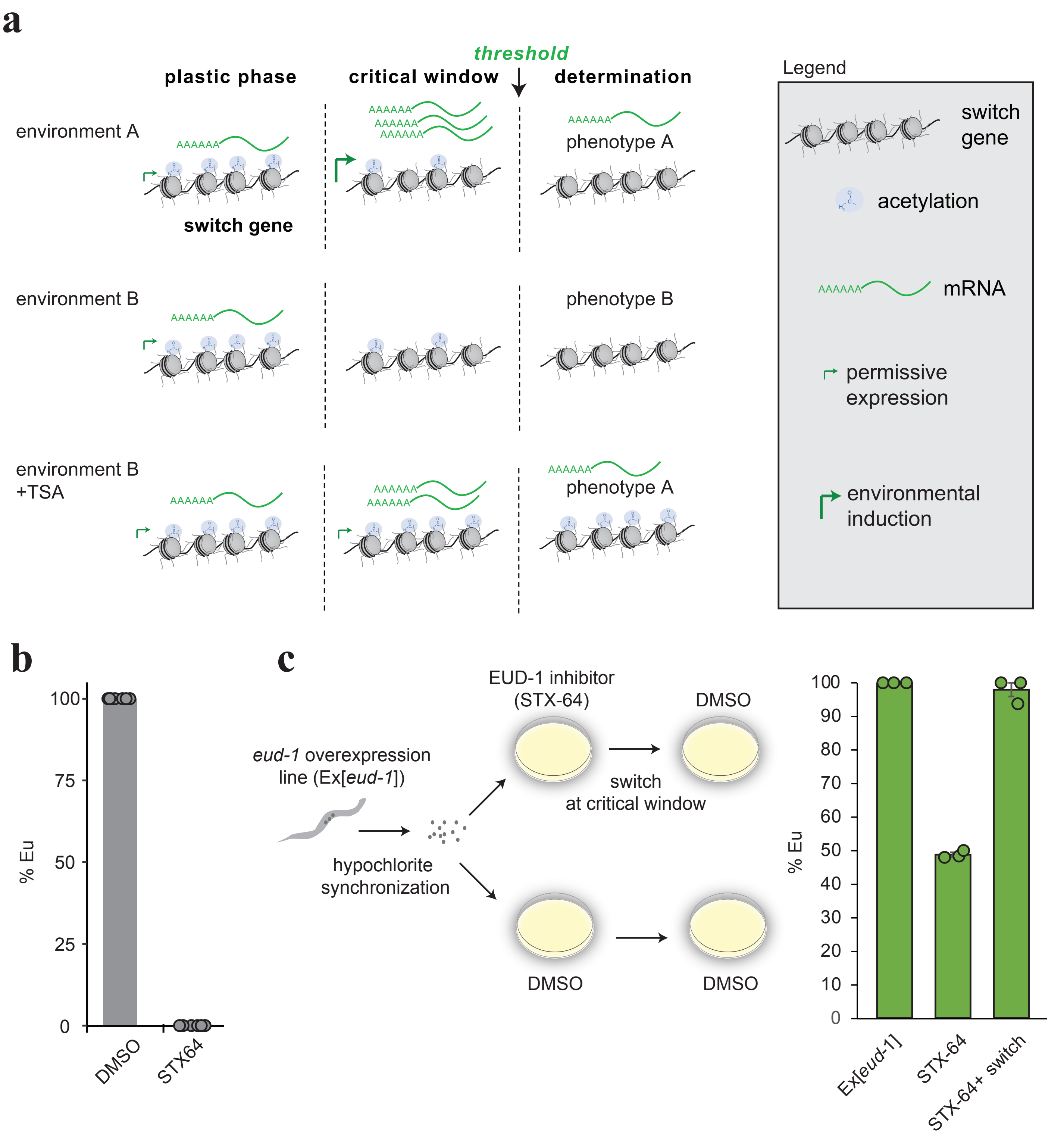

Supplementary Figure 7: Model for establishment of plasticity during the critical window. a, Plasticity is established by environment-independent H4K5/12ac and permissive transcription during the critical window. Ultimately H4K5/12 is deacetylated leading toward repression of switch genes and the end of the critical window. When TSA is added in liquid culture, the fixed juvenile chromatin state of H4K5/12ac prevents switch gene repression. **b**, STX-64 induces the St phenotype at 1 µg/ml. n=6 STX64, 7 DMSO, with at least 20 worms per replicate. **c**, Switching between STX-64 plates and DMSO plates at 48 hours 'rescues' the Ex[eud-1] phenotype=3 biologically independent worm populations. Error bars represent S.E.M. Source data are provided as a Source Data file.

Source Data: Ponceau Red Stains in western blots (Supplementary Fig. 4d)

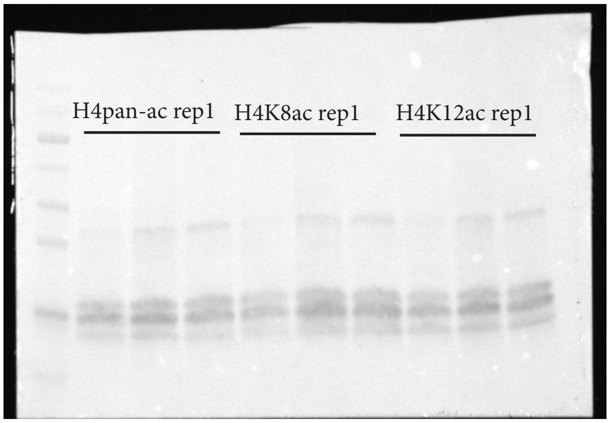

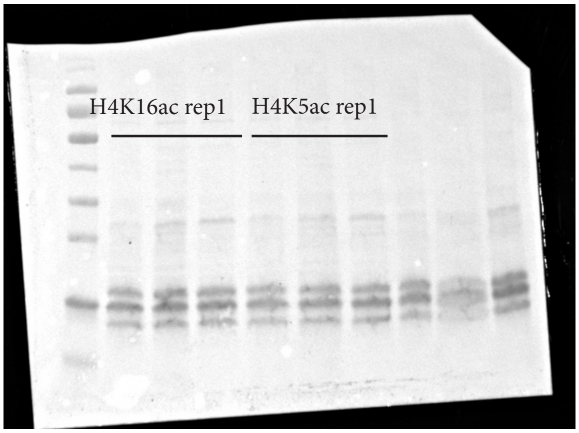

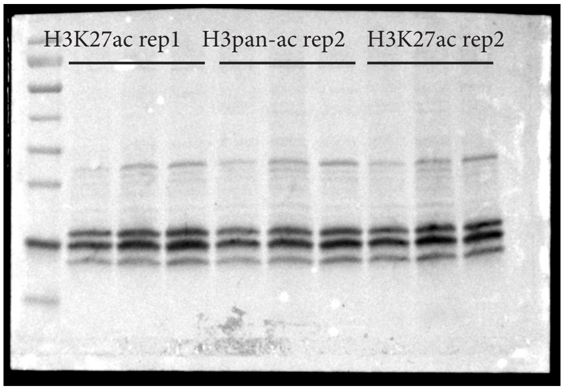

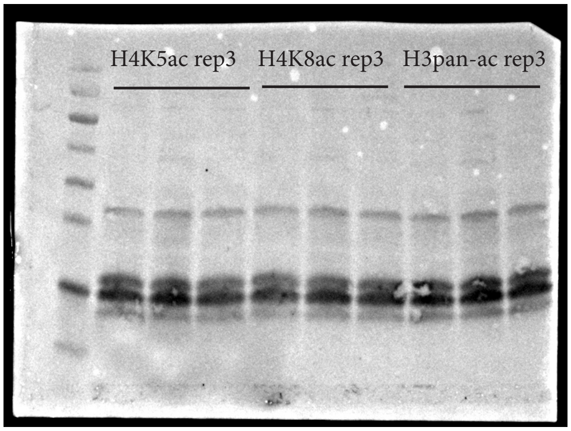

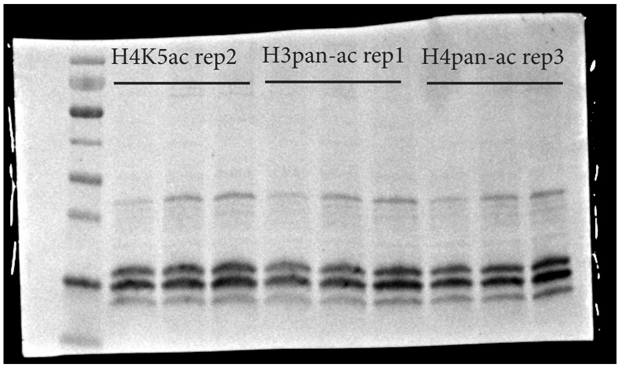

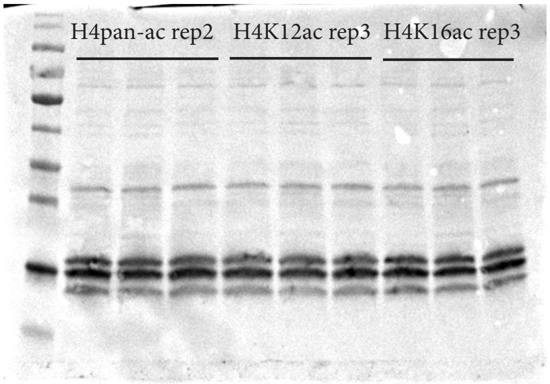

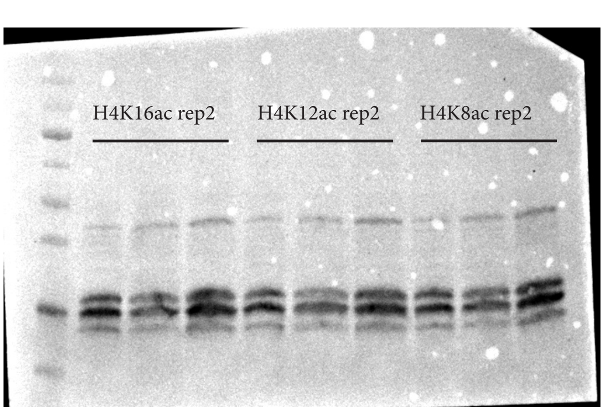

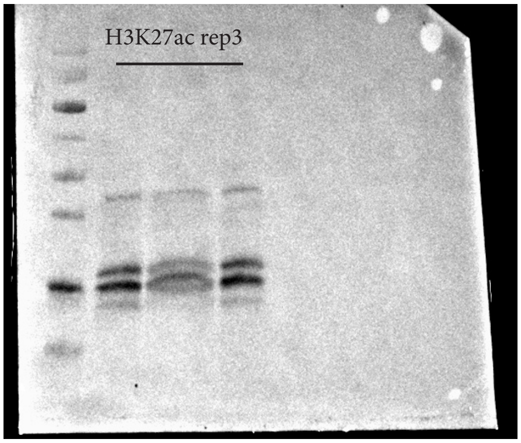

Source Data: Western Blots (Supplementary Fig. 4d). Note, strips of nitrocellulose membrane corresponding to biological replicates of DMSO, Butyrate and TSA were cut after ponceau staining and probed separately for each antibody to reduce primary-Ab incubation volume.

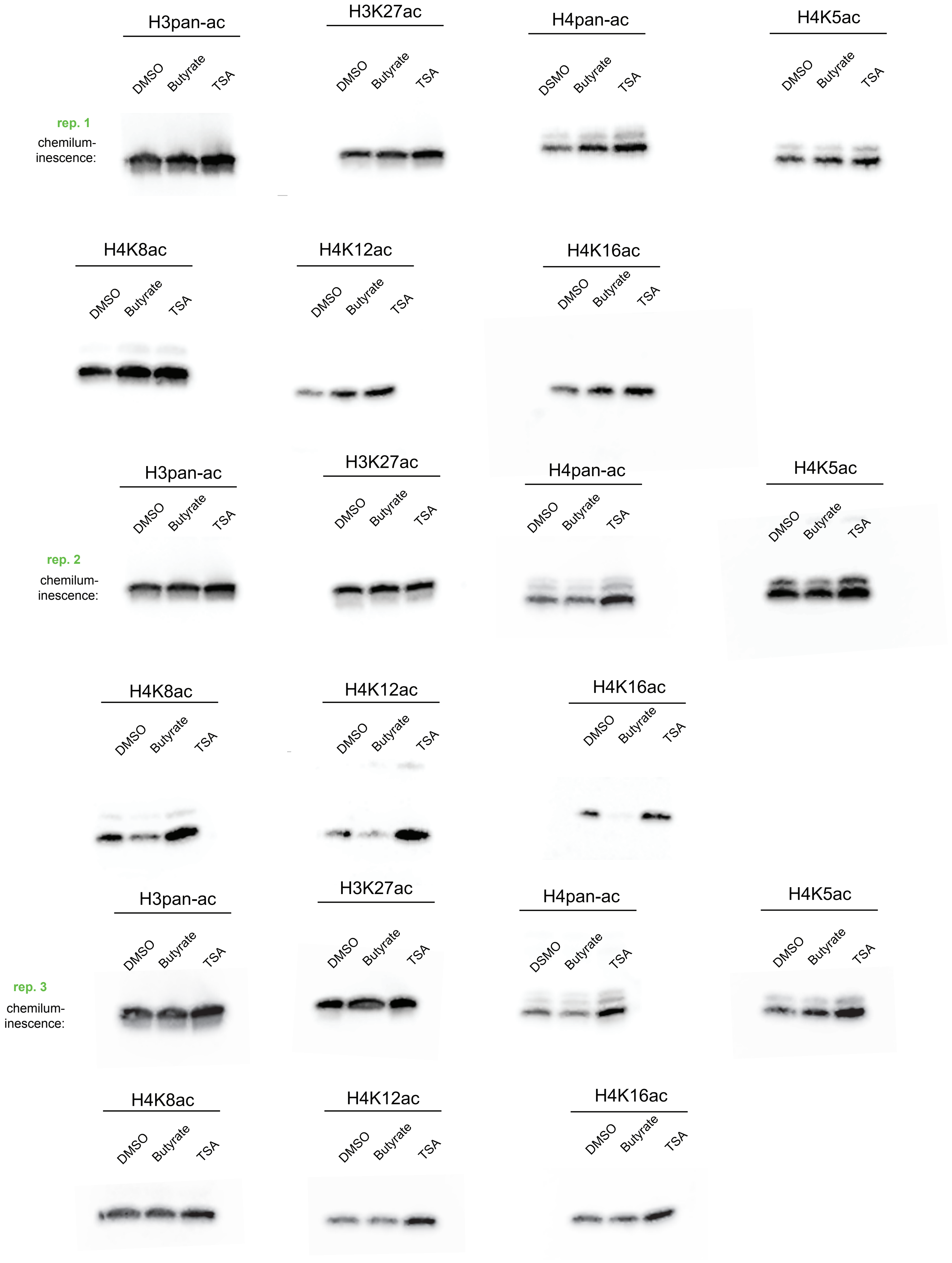

Supplement: Supplementary file 1 — Supplementary Information [file 41467_2023_37734_MOESM1_ESM.pdf]
