# Supplementary material for: Histone 4 lysine 5/12 acetylation enables developmental plasticity of Pristionchus mouth form
Source: Nat Commun. 2023 Apr 13;14:2095. doi: 10.1038/s41467-023-37734-z (PMC10102330; doi:10.1038/s41467-023-37734-z)
Supplement: Supplementary file 3 — Description of Additional Supplementary Files [file 41467_2023_37734_MOESM3_ESM.pdf]

Description of Additional Supplementary Files

File Name: Supplementary Data 1
Description: Antibodies and primers

File Name: Supplementary Data 2
Description: Raw data of LC-MS/MS acetylated sites (non-redundant).

File Name: Supplementary Data 3
Description: acetyl-ChIP-seq mapping statistics and MACS2 peaks
