# Supplementary material for: Histone 4 lysine 5/12 acetylation enables developmental plasticity of Pristionchus mouth form
Source: Nat Commun. 2023 Apr 13;14:2095. doi: 10.1038/s41467-023-37734-z (PMC10102330; doi:10.1038/s41467-023-37734-z)
Supplement: Supplementary file 7 — Reporting Summary [file 41467_2023_37734_MOESM7_ESM.pdf]

nature portfolio

Corresponding author(s): Ralf J. Sommer

Last updated by author(s): Mar 6, 2023

Reporting Summary

Nature Portfolio wishes to improve the reproducibility of the work that we publish. This form provides structure for consistency and transparency in reporting. For further information on Nature Portfolio policies, see our Editorial Policies and the Editorial Policy Checklist.

Statistics

For all statistical analyses, confirm that the following items are present in the figure legend, table legend, main text, or Methods section.

n/a	Confirmed	
☐	☒	The exact sample size (*n*) for each experimental group/condition, given as a discrete number and unit of measurement
☐	☒	A statement on whether measurements were taken from distinct samples or whether the same sample was measured repeatedly
☐	☒	The statistical test(s) used AND whether they are one- or two-sided<br>*Only common tests should be described solely by name; describe more complex techniques in the Methods section.*
☐	☒	A description of all covariates tested
☐	☒	A description of any assumptions or corrections, such as tests of normality and adjustment for multiple comparisons
☐	☒	A full description of the statistical parameters including central tendency (e.g. means) or other basic estimates (e.g. regression coefficient) AND variation (e.g. standard deviation) or associated estimates of uncertainty (e.g. confidence intervals)
☐	☒	For null hypothesis testing, the test statistic (e.g. *F*, *t*, *r*) with confidence intervals, effect sizes, degrees of freedom and *P* value noted<br>*Give P values as exact values whenever suitable.*
☐	☒	For Bayesian analysis, information on the choice of priors and Markov chain Monte Carlo settings
☐	☒	For hierarchical and complex designs, identification of the appropriate level for tests and full reporting of outcomes
☐	☒	Estimates of effect sizes (e.g. Cohen's *d*, Pearson's *r*), indicating how they were calculated

Our web collection on statistics for biologists contains articles on many of the points above.

Software and code

Policy information about availability of computer code

Data collection	no software was used for data collection
Data analysis	All code is included in the methods section, which uses standard and open-source bioinformatic software. For proteomic analysis, we used MaxQuant software version 1.5.2.8. For Chip-Seq analysis we used HOMER V4 and MACS2 software programs.

For manuscripts utilizing custom algorithms or software that are central to the research but not yet described in published literature, software must be made available to editors and reviewers. We strongly encourage code deposition in a community repository (e.g. GitHub). See the Nature Portfolio guidelines for submitting code & software for further information.

Data

Policy information about availability of data

All manuscripts must include a data availability statement. This statement should provide the following information, where applicable:

- Accession codes, unique identifiers, or web links for publicly available datasets
- A description of any restrictions on data availability
- For clinical datasets or third party data, please ensure that the statement adheres to our policy

ChIP-seq datasets generated during this study are available at the National Center for Biotechnology Information Sequence Read Archive (NCBI SRA) data base under the accession PRJNA628502, and can be accessed with the following link: https://www.ncbi.nlm.nih.gov/sra/PRJNA628502. The mass spectrometry

proteomics data have been deposited to the ProteomeXchange Consortium via the PRIDE partner repository with the dataset identifier PXD018940 (https://www.ebi.ac.uk/pride/archive/projects/PXD018940). For mapping ChIP-seq reads we used the 'El Paco' genome publicly available from Pristionchus.org.

Human research participants

Policy information about studies involving human research participants and Sex and Gender in Research.

Reporting on sex and gender	no human research participants were used in this study
Population characteristics	no human research participants were used in this study
Recruitment	no human research participants were used in this study
Ethics oversight	no human research participants were used in this study

Note that full information on the approval of the study protocol must also be provided in the manuscript.

Field-specific reporting

Please select the one below that is the best fit for your research. If you are not sure, read the appropriate sections before making your selection.

☒ Life sciences ☐ Behavioural & social sciences ☐ Ecological, evolutionary & environmental sciences

For a reference copy of the document with all sections, see nature.com/documents/nr-reporting-summary-flat.pdf

Life sciences study design

All studies must disclose on these points even when the disclosure is negative.

Sample size	Sample sizes were chosen depending on the method being used and effect size, in order to provide sufficient statistical power.
Data exclusions	No data were excluded from this study
Replication	Different numbers of replicates were performed depending on the effect size of the experimental difference. All results were viewed as valid and incorporated in the final result. Replication reported throughout the manuscript was of biologically independent worm populations. Geometric Morphometrics, Western blots, inhibitor assays, ChIP-experiments, and staging were done at independent times with different worm populations. RT-qPCR of eud-1 in the presence and absence of TSA was performed from multiple biological populations derived from (and normalized to) the same original population of eggs.
Randomization	Randomization was not relevant to the sample sizes in this study, which were of large effect size and with small numbers of sample populations. There was nevertheless some de facto randomization; for example the location of flasks and culture plates was random with respect to sample.
Blinding	Blinding was done for mouth-form phenotyping. Sample IDs on microscope slides were covered with tape during screening, and only revealed after scoring phenotype. Blinding was also done during placement of landmarks for Geometric Morphometrics. For molecular biology and biochemical experiments (i.e., western blots, RT-qPCR), blinding is not standard. These experiments were also repeated in independent replicates, mitigating the concern of bias from single experiments.

Reporting for specific materials, systems and methods

We require information from authors about some types of materials, experimental systems and methods used in many studies. Here, indicate whether each material, system or method listed is relevant to your study. If you are not sure if a list item applies to your research, read the appropriate section before selecting a response.

Materials & experimental systems

n/a	Involved in the study	
☐	☒	Antibodies
☒	☐	Eukaryotic cell lines
☒	☐	Palaeontology and archaeology
☐	☒	Animals and other organisms
☒	☐	Clinical data
☒	☐	Dual use research of concern

Methods

n/a	Involved in the study	
☐	☒	ChIP-seq
☒	☐	Flow cytometry
☒	☐	MRI-based neuroimaging

Antibodies

Antibodies used	All antibodies used in this study are reported in Extended Data Table 1:<br>epitope company animal/type cat # lot# dilution<br>H4panAc ActiveMotif rabbit IgG 39026 1518004 1:1,000<br>H4K5Ac Diagenode rabbit polyclonal C15410025 A1456D 1:2,000<br>H4K8Ac Diagenode rabbit polyclonal C15410103 A157-004 1:1,000<br>H4K12Ac Diagenode rabbit polyclonal C15410331 A2439P 1:1,000 (used in worm experiments)<br>H4K12Ac Millipore rabbit monoclonal 04-119-S 3766681 1:2,000 (used in fly experiments)<br>H4K16ac Merck rabbit polyclonal 07-329 3170793 1:1,000<br>H3panAc ActiveMotif rabbit polyclonal 39139 2851008 1:2,000<br>H3K27ac Diagenode rabbit polyclonal C15410174 1:1,000<br>secondary antibody<br>IgG-HRP Cell Signalling Anti-rabbit 7074S 28 1:2,000<br><br>*Note, all antibodies used in fly were at 1:2,000
Validation	All antibodies used are commercially available and the corresponding websites include standard ChIP and western blot validation. Tested species reactivities for primary antibodies are listed below:<br><br>H4panAc ActiveMotif: Human, mouse, wide range predicted<br>H4K5Ac Diagenode: Human, mouse, wide range expected<br>H4K8Ac Diagenode: Human, mouse<br>H4K12Ac Diagenode: Human, mouse, wide range expected<br>H4K12Ac Millipore rabbit monoclonal: Reacts with Human, wide range of cross-reactivity expected based on sequence homology.<br>H4K16ac Merck: Human, mouse, rat, but broad species cross-reactivity is expected.<br>H3panAc ActiveMotif: Human, wide range predicted.<br>H3K27ac Diagenode: Human, mouse, rat, pig: positive. Other species: not tested.<br><br>Antibodies have not been previously tested in Pristionchus, but histones and histone post-translational modifications are among the most conserved proteins and modifications across all Eukarya.

Animals and other research organisms

Policy information about studies involving animals; ARRIVE guidelines recommended for reporting animal research, and Sex and Gender in Research

Laboratory animals	Pristionchus pacificus (strain ID: PS312), Caenorhabditis elegans (strain ID: N2), and Drosophila melanogaster (strain ID: w1118 (Bloomington Drosophila Stock Center #3605). The age of the animals are indicated in each experiment, and are often a variable in the experiment.
Wild animals	no wild animals were used in this study.
Reporting on sex	nematodes (P. pacificus and C. elegans) populations were maintained at >95% hermaphrodites. Sex of Drosophila was not measured but assumed to be 50/50.
Field-collected samples	No field collected samples were used in this study.
Ethics oversight	ethical approval was not required for invertebrates used in this study

Note that full information on the approval of the study protocol must also be provided in the manuscript.

ChIP-seq

Data deposition

☒ Confirm that both raw and final processed data have been deposited in a public database such as GEO.

☒ Confirm that you have deposited or provided access to graph files (e.g. BED files) for the called peaks.

Data access links<br>*May remain private before publication.*	PRJNA628502
Files in database submission	H4K5ac rep1-2, H4K8ac rep1-2, H4K12ac rep1-2, H4K16ac rep1-2, H3K27me3 rep1-2, H3pan-ac rep1-2, H4pan-ac rep1-2,
Genome browser session<br>(e.g. UCSC)	no longer applicable.

Methodology

Replicates	2
Sequencing depth	pair-end, >7 million per library
Antibodies	Same as above (see antibodies)
Peak calling parameters	default with macs2 and Homer 'annotatePeaks' using input as control
Data quality	Quality score > 30, and Homer enrichment > 1%
Software	Homer software suite and macs2
